# Fahr’s Disease With Late Onset: A Case Report

**DOI:** 10.7759/cureus.23316

**Published:** 2022-03-19

**Authors:** Saba Iqbal, Mahmoud Nassar, Howard Chung, Tanveer Shaukat, Jeffrey E Penny, Vincent Rizzo

**Affiliations:** 1 Internal Medicine, Icahn School of Medicine at Mount Sinai/NYC (New York City) Health+Hospitals/Queens, New York, USA; 2 Internal Medicine, St. George's University, New York, USA

**Keywords:** geriatric patient, idiopathic basal ganglia calcification (ibgc), rare genetic diseases, basal ganglia disease, rare cause of altered mental status, fahr’s disease or fahr’s syndrome

## Abstract

Fahr's disease is a rare genetically dominant disease. It is characterized by the idiopathic deposition of calcium in the basal ganglia and cerebral cortex. The condition may cause motor impairment, impaired muscle tone, dementia, seizures, impairment of eye movements, speech, abnormal hand movements, cognitive impairment, and ataxia. The thalamus, white matter, and basal ganglia can be involved. A 77-year-old man with multiple comorbidities presented with a complaint of increasing confusion, altered mental status, dystonia, tremor, and hallucinations. The patient's daughter reported that he sounded confused and inappropriate in his speech. A computerized tomography (CT) scan of the head without contrast revealed a "dense calcification of the dentate nuclei and the basal ganglia" and "subcortical calcification of the frontal and occipital lobes." The patient was diagnosed with late-onset Fahr's disease. Fahr's disease is caused by idiopathic calcification of the bilateral basal ganglia. A wide variety of symptoms are associated with this condition. Fahr's disease should be considered in the differential diagnosis in geriatric patients suffering from cognitive impairment and movement disorders.

## Introduction

Fahr's disease is a rare genetically dominant disease, and it is characterized by the idiopathic deposition of calcium in the basal ganglia and cerebral cortex [1]. It may cause motor function impairment, impaired muscle tone, dementia, seizure, impaired eye movements, speech, abnormal writhing hand movements, cognitive impairment, and ataxia [2]. In this disease, calcification can involve the thalamus, white matter, and basal ganglia, and symptoms range from symptomatic to various neurological conditions [3]. Fahr's disease also presents with Parkinson-like features like a mask-like face, shuffling gait, muscle rigidity, and resting pin-rolling tremors of hands. These symptoms appear in the advanced stage of the disease. The usual age of onset is from 40 to 50, but it can occur at any age, including childhood. Computerized tomography (CT) scan of the brain is the most effective technique for detecting and localizing intracranial calcifications [4]. Other names for Fahr's disease are primary familial brain calcifications and familial basal ganglia calcifications.

## Case presentation

**Figure 1:**
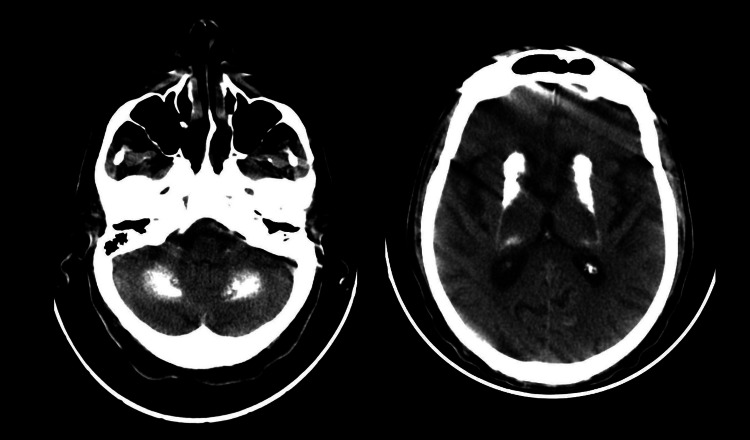
A CT scan of the head without contrast showed dense calcification of the dentate nuclei and the basal ganglia and subcortical calcification of the frontal and occipital lobes

A 77-year-old male with a past medical history of post-traumatic stress disorder (PTSD), multiple cerebrovascular accidents (CVAs), chronic kidney disease (CKD) stage 3b, hypertension, hyperlipidemia, type 2 diabetes mellitus, diabetic neuropathy, and benign prostatic hyperplasia (BPH) presented with a complaint of increasing confusion, altered mental status, dystonia, tremor, and hallucinations. The family did not have a history of neurological disease. According to the patient's daughter, the patient sounded confused and inappropriate in his speech. The patient was admitted for evaluation of altered mental status. The patient was alert and oriented to person, place, time, and situation in the emergency department, with shudder while neurologically intact. The patient was aggressive, agitated, and began wandering the hallways, prompting placement of 1:1 observation. Psychiatry was consulted for evaluation. A CT scan of the head without contrast (Figure 1) had the impression of "dense calcification of the dentate nuclei and the basal ganglia" and "subcortical calcification of the frontal and occipital lobes" or primary familial brain calcification, highly suggestive of Fahr's syndrome. The patient's phosphorus level was 3.5 mg/dl (normal level: 2.5-4.5 mg/dl). Parathyroid hormone (PTH) intact was 39 pg/ml (normal level: 15-65 pg/ml), and calcium level was 9.7 mg/dl (normal level: 8.4-10.5 mg/dl). The vitamin D 25-hydroxy concentration was 39.4 ng/ml (normal level: 30-60 ng/ml).

The home medication list includes Plavix 75 mg, atorvastatin 20 mg, tamsulosin 0.4 mg, Lamictal 25 mg, trazodone 50 mg, gabapentin 200 mg, and risperidone 0.25 mg two times a day (bid). The patient was prescribed Sinemet for dystonia and tremor of the hand and tongue upon admission. Furthermore, lamotrigine was reduced from 75 to 25 mg, and trazodone was reduced from 150 to 50 mg. The patient then became more awake, and his tremor and dystonia became evident. Before being transferred to a medicine inpatient unit, the patient received Haldol, Ativan, and midazolam in the emergency room. The patient's blood analysis revealed hypophosphatemia, which was corrected with the assistance feed. The patient's family refused genetic testing and further imaging for the patient. The patient's daughter was notified and counseled that there is no medical cure for Fahr's disease, and the focus of care was then on symptomatic control and psychiatric management. Although the patient's altered mental status improved on day 14, he was often confused and mumbling requiring redirection and wandering in the unit. After being hospitalized for five weeks, he was transferred to a long-term care facility.

## Discussion

Fahr's disease, also known as idiopathic basal ganglia calcification, is a rare neurodegenerative disorder caused by the accumulation of calcium in the basal ganglia. Fahr's syndrome and Fahr's disease are two different conditions that differ in their etiology, locations of lesions, prognosis, and treatments. Various disorders can cause intracranial calcifications, such as metabolic, infectious, congenital, vascular, and neoplastic conditions. Hypoparathyroidism is the most common endocrine disorder associated with intracranial disorders. Toxoplasmosis, rubella, cytomegalovirus, HIV, tuberculosis, and syphilis are some of the most common infectious diseases that cause intracranial calcifications. An autoimmune condition such as systemic lupus erythematosus (SLE) may also cause intracranial calcification [5]. Fahr's syndrome can be diagnosed when basal ganglia calcifications are secondary to known causes and associated presentations are observed. Fahr's disease should be diagnosed based on clinical presentation, imaging evidence, and associated family history after excluding other intracranial calcification causes. Primary familial brain calcification, also known as familial basal ganglia calcification, is inherited autosomal dominant. Several gene mutation sites are related to familial basal ganglia calcification: PDGFB, PDGFRB, SLC20A2, and XPR1 [6]. In the case of positive family history, comprehensive genetic testing and genetic counseling should be provided.

The disease usually manifests between the ages of 40 and 60, primarily after the age of 30 [7]. The disease progresses slowly and is more common in males than in females. Based on the previous studies, the incidence of intracranial calcifications in the basal ganglia detected by CT head indicates that Fahr's syndrome is approximately between 0.24% and 1.64% [8]. Fahr's disease is characterized by bilateral symmetrical calcification of the basal ganglia. The globus pallidus is the most involved basal ganglion. Calcium deposits can also be found in dentate nuclei, thalami, and other deep cortical structures [9]. Fahr's disease is characterized by movement disorders, cognitive impairments, neuropsychiatric symptoms (such as hallucination, delusion, anxiety, irritability, and aggression), mood disorders (such as depression and bipolar disorder), and personality disorders [5,10].

The most common manifestation of Fahr's disease is movement disorders. Parkinsonism is the most common movement disorder among Fahr's disease patients, accounting for more than half of their clinical presentations [11]. Other movement disorders such as chorea, tremor, dystonia athetosis, and orofacial dyskinesia can also be found in Fahr's disease patients. Other somatic symptoms such as seizures, headache, vertigo, stroke, syncope, ataxia, dysarthria, tremor, and orthostatic hypotension are also reported from case studies.

There is one case report with late-onset Fahr's disease and basal ganglia calcification [12]. By publishing this case, Fahr's disease has gained attention as a diagnosis to be considered in the differential diagnosis of geriatric patients with cognitive impairment, such as Alzheimer's, and movement disorders, such as Parkinson's. Symptomatic treatments for parkinsonism and mood disorders include levodopa and antipsychotics. Muscle relaxants and antiepileptic drugs are also used to treat dystonia and seizures. Treatment of underlying hypoparathyroidism may improve neuropsychiatric symptoms. Currently, no effective treatment can limit or slow down calcification in the basal ganglia.

## Conclusions

Fahr's disease results from idiopathic calcification of the bilateral basal ganglia. There is no specific treatment for Fahr's disease. Fahr's disease should be considered in the differential diagnosis in geriatric patients suffering from cognitive impairment and movement disorders.
